# Pioneering Augmented and Mixed Reality in Cranial Surgery: The First Latin American Experience

**DOI:** 10.3390/brainsci14101025

**Published:** 2024-10-16

**Authors:** Alberto Ramírez Romero, Andrea Rebeca Rodríguez Herrera, José Francisco Sánchez Cuellar, Raúl Enrique Cevallos Delgado, Edith Elizabeth Ochoa Martínez

**Affiliations:** 1 Neurosurgeon Hospital Ángeles Universidad, Mexico City 03330, Mexico; josefrancisco5678@gmail.com (J.F.S.C.); cevallosneurocirugia@gmail.com (R.E.C.D.); 2 Neurology Resident CMN 20 de Noviembre, ISSSTE, UNAM, Mexico City 34079, Mexico; beckyrherrera2318@gmail.com; 3 Neuroanesthesiology Hospital Ángeles Universidad, Mexico City 03330, Mexico; ochoa.martinez.edithe@gmail.com

**Keywords:** virtual reality, augmented reality, mixed reality, cranial surgery, neurosurgery, neuro-oncology

## Abstract

Introduction: Augmented reality (AR) and mixed reality (MR) technologies have revolutionized cranial neurosurgery by overlaying digital information onto the surgical field, enhancing visualization, precision, and training. These technologies enable the real-time integration of preoperative imaging data, aiding in better decision-making and reducing operative risks. Despite challenges such as cost and specialized training needs, AR and MR offer significant benefits, including improved surgical outcomes and personalized surgical plans based on individual patient anatomy. Materials and Methods: This study describes three intracranial surgeries using AR and MR technologies at Hospital Ángeles Universidad, Mexico City, in 2023. Surgeries were performed with VisAR software 3 version and Microsoft HoloLens 2, transforming DICOM images into 3D models. Preoperative MRI and CT scans facilitated planning, and radiopaque tags ensured accurate image registration during surgery. Postoperative outcomes were assessed through clinical and imaging follow-up. Results: Three intracranial surgeries were performed with AR and MR assistance, resulting in successful outcomes with minimal postoperative complications. Case 1 achieved 80% tumor resection, Case 2 achieved near-total tumor resection, and Case 3 achieved complete lesion resection. All patients experienced significant symptom relief and favorable recoveries, demonstrating the precision and effectiveness of AR and MR in cranial surgery. Conclusions: This study demonstrates the successful use of AR and MR in cranial surgery, enhancing precision and clinical outcomes. Despite challenges like training and costs, these technologies offer significant benefits. Future research should focus on long-term outcomes and broader applications to validate their efficacy and cost-effectiveness in neurosurgery.

## 1. Introduction

Augmented reality (AR) in surgery represents a cutting-edge technology that has rapidly evolved in recent years, offering significant advancements in visualization, precision, and surgical training. AR overlays digital information onto the surgeon’s field of view, providing real-time guidance during complex procedures such as tumor resections or joint replacements. Mixed reality (MR), which integrates virtual and physical environments, further enhances this capability by allowing seamless interaction between the digital and real worlds [1,2,3].

In cranial neurosurgery, AR and MR technologies have revolutionized surgical navigation and planning, leading to more precise outcomes and reduced invasiveness. These technologies enable surgeons to integrate preoperative imaging data directly into their line of sight, aiding in better decision-making and reducing operative risks by overlaying critical information, like tumor margins or neural pathways, during the procedure [2,3,4]. Furthermore, AR facilitates realistic surgical simulations, allowing trainees to practice in a controlled environment before performing actual surgeries, thus improving surgical skills and patient safety [4].

The integration of AR and MR into clinical workflows presents several considerations and challenges. These include cost-effectiveness, the need for specialized training for medical staff, the devices and software required, and regulatory considerations. Despite these challenges, the potential benefits of these technologies are immense, including enhanced precision, improved surgical outcomes, and the ability to provide personalized surgical plans based on individual patient anatomy [5,6,7].

Different devices construct a virtual environment, only VisAR (Novarad, Provo, UT, USA), a software developed 8years ago and the most complete program, was the first device to obtain FDA approval for surgery planification. VisAR is a software that works with HoloLens 2 (Microsoft, Redmond, WA, USA). It transforms Digital Imaging and Communications in Medicine (DICOM) images/studies into three-dimensional virtual images that are superimposed directly on to the patient using HoloLens 2, which can be manipulated by voice commands from the surgeons. There are self-adhesive tags, printed with radiopaque ink for image registration. The tags are placed on the patient around the surgical site, either on the skin or attached to the bones [8,9]. This technique has an accuracy of 2–3 mm. The anatomy and the insertion pathway are continuously visible to the surgeon throughout the procedure because the hologram is superimposed on the surgical site [1,9,10]. The surgeon needs to practice and undertake planification before the procedure to match the images with the software.

In this paper, we present our pioneering experience with AR and MR in cranial neurosurgery in Latin America (LATAM). Since 2023, our team has been the first in the region to introduce this technology into intracranial and spinal surgeries. We provide detailed accounts of three intracranial surgery cases with varying degrees of complexity, all assisted by AR and MR, demonstrating the practical applications and benefits of these technologies in real surgical scenarios.

## 2. Materials and Methods

### 2.1. Study Design

This study is a descriptive analysis of three intracranial surgery cases performed with the assistance of augmented reality (AR) and mixed reality (MR) technologies. The surgeries took place between January 2023 and December 2023 at Hospital Ángeles Universidad in Mexico City, Mexico. The primary objective was to evaluate the feasibility, accuracy, and clinical outcomes of AR- and MR-assisted cranial surgeries.

### 2.2. Patient Selection

Three patients, selected based on their clinical presentation and radiological findings, underwent AR- and MR-assisted surgeries. The inclusion criteria were patients diagnosed with intracranial lesions requiring surgical intervention, without contraindications for AR/MR technology. The exclusion criteria were patients with severe systemic diseases or allergies to the materials used in the procedure.

### 2.3. Equipment and Software

The surgeries were performed using VisAR software 3 version (Novarad, Provo, UT, USA) integrated with Microsoft HoloLens 2 (Microsoft, Redmond, WA, USA). VisAR transforms Digital Imaging and Communications in Medicine (DICOM) images into three-dimensional (3D) virtual images that are superimposed onto the patient’s anatomy during surgery. Radiopaque self-adhesive tags were used for image registration, providing an accuracy of 2–3 mm.

### 2.4. Preoperative Planning

1. Imaging Acquisition: Preoperative MRI and CT scans were obtained for each patient, ensuring high-quality imaging data for 3D reconstruction.
2. Image Processing: DICOM images were processed using VisAR software to create 3D virtual models of the patient’s anatomy, highlighting the lesions and surrounding critical structures.
3. Registration: Radiopaque tags were placed around the surgical site on the patient’s skin or bones to facilitate the accurate registration of virtual images with real-time anatomy during surgery.

### 2.5. Surgical Procedure

1. Setup: Patients were positioned according to standard neurosurgical protocols for the specific cranial approach. The HoloLens 2 device was calibrated, and the AR system was configured.
2. Navigation: Surgeons wore the HoloLens 2 device, which superimposed the 3D virtual images onto the patient’s anatomy. Voice commands were used to manipulate the images.
3. Surgical Intervention: The surgeries were performed using standard neurosurgical techniques with real-time AR guidance. The surgeon’s field of view included critical information overlaid on the patient’s anatomy, enhancing precision in lesion localization and resection.

## 3. Results

### 3.1. Case 1

A 62-year-old female with a history of arterial hypertension and venous thrombosis, treated with rivaroxaban 10 mg and clopidogrel 75 mg, presented with a month-long history of hemicranial headache. The headache progressively intensified, worsened with postural changes, and was accompanied by nausea, fever, insomnia, dizziness, and a predominant right lateral gait disturbance.

**Figure 1:**
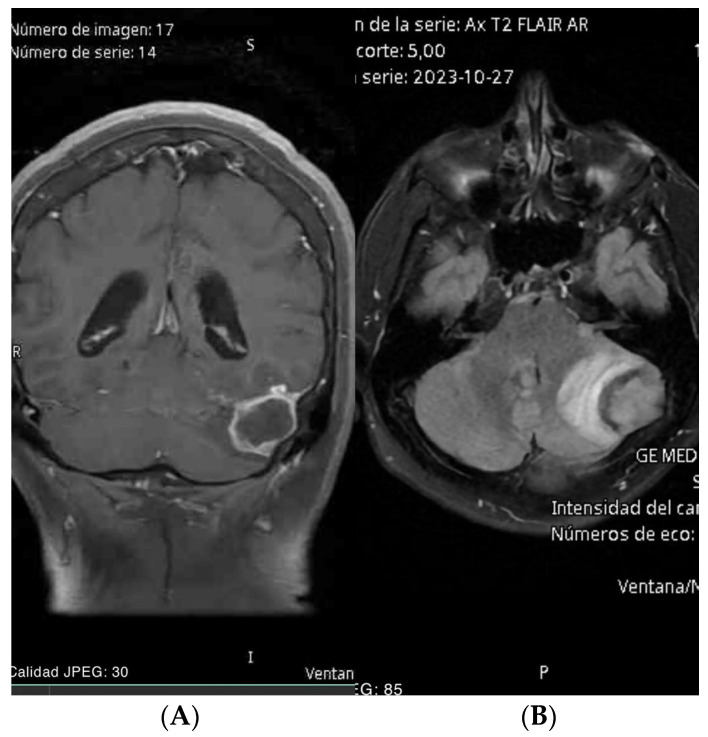
(**A**) Brain MRI coronal view: extra-axial infratentorial lesion adjacent to the left cerebellar hemisphere with ring enhancement with a volume effect. (**B**) Brain MRI axial view FLAIR: extra-axial infratentorial lesion adjacent to the left cerebellar hemisphere with a volume effect.

Upon consultation, a contrast-enhanced MRI of the brain was requested. The imaging revealed an extra-axial infratentorial lesion adjacent to the left cerebellar hemisphere, causing a mass effect with displacement of structures, edema, and compression of the fourth ventricle leading to its dilation (Figure 1A,B). The differential diagnosis included an unknown tumor versus an abscess.

**Figure 2:**
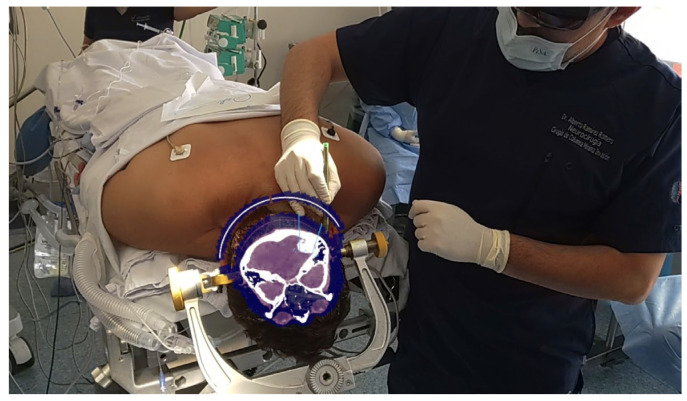
Target placement with AR, where the patient’s CT scan can be observed in the coronal view.

A retrosigmoid approach with opening of the pontine and medullary brain cisterns was performed, achieving an 80% subtotal resection of the tumor with the assistance of augmented reality (AR) navigation (Figure 2). The postoperative period was uneventful, and the patient was free of headaches and other symptoms.

**Figure 3:**
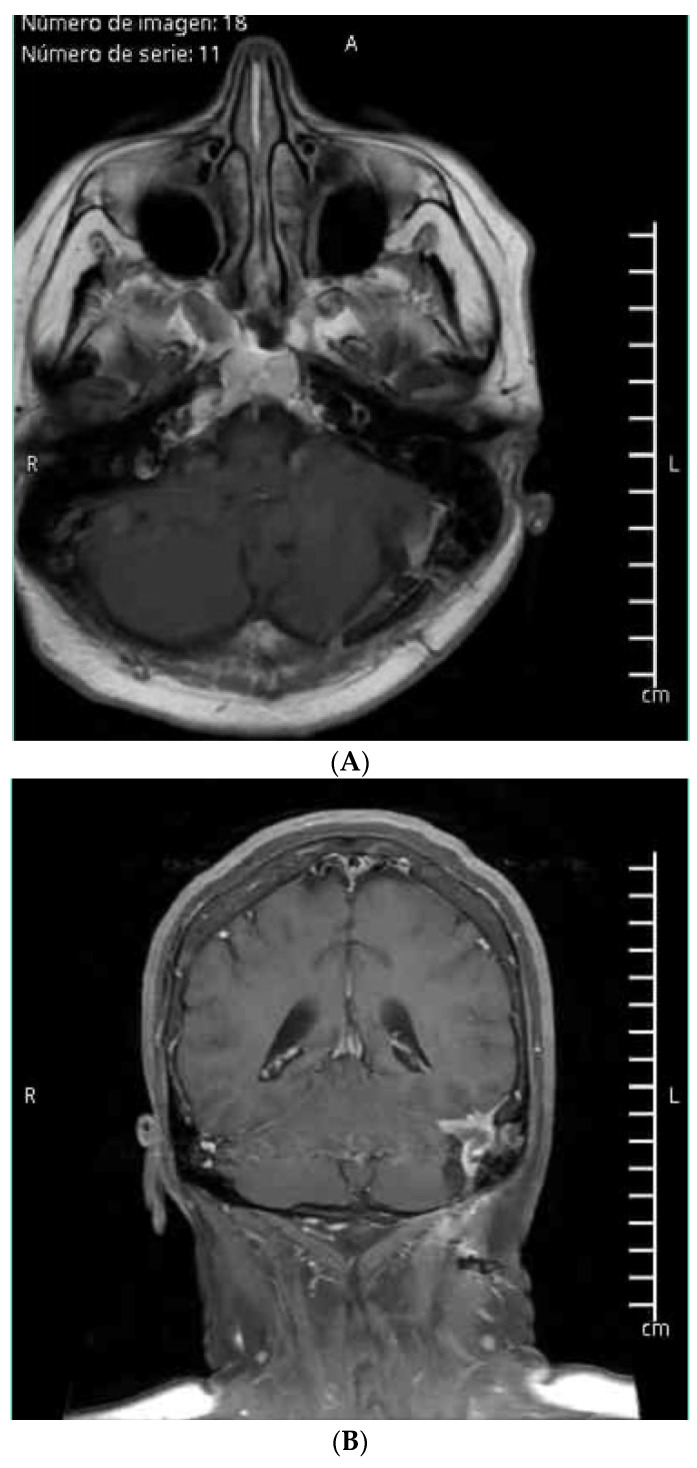
(**A**) MRI axial view post-procedure. (**B**) MRI coronal view post-surgery.

Postoperative MRI showed surgical changes and a residual image of the meningioma in intimate contact with the transverse sinus, without alteration of its signal (Figure 3A,B). Histopathological analysis identified the lesion as a psammomatous meningioma.

### 3.2. Case 2

**Figure 4:**
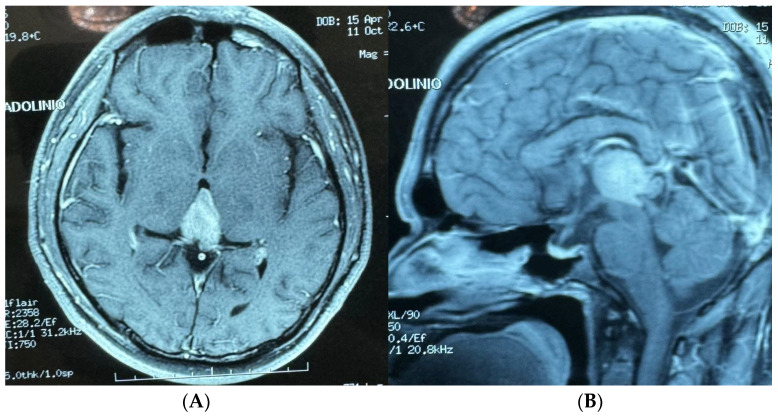
(**A**) Brain MRI axial view with enhancement in inter pulvinar pineal in the 3rd ventricle. (**B**) Brain MRI sagittal view with enhancement in inter pulvinar pineal in the 3rd ventricle.

A 30-year-old male with no significant medical history presented to the Emergency Room with a sudden headache and concerning symptoms. Initial evaluation included a CT scan of the brain, followed by a contrast-enhanced MRI, which revealed obstructive hydrocephalus. To address this, a right precoronal peritoneal ventricle bypass valve was initially placed. During this procedure, a tumor was identified in the pineal region, extending into the third ventricle and tentorial notch (Figure 4A,B).

**Figure 5:**
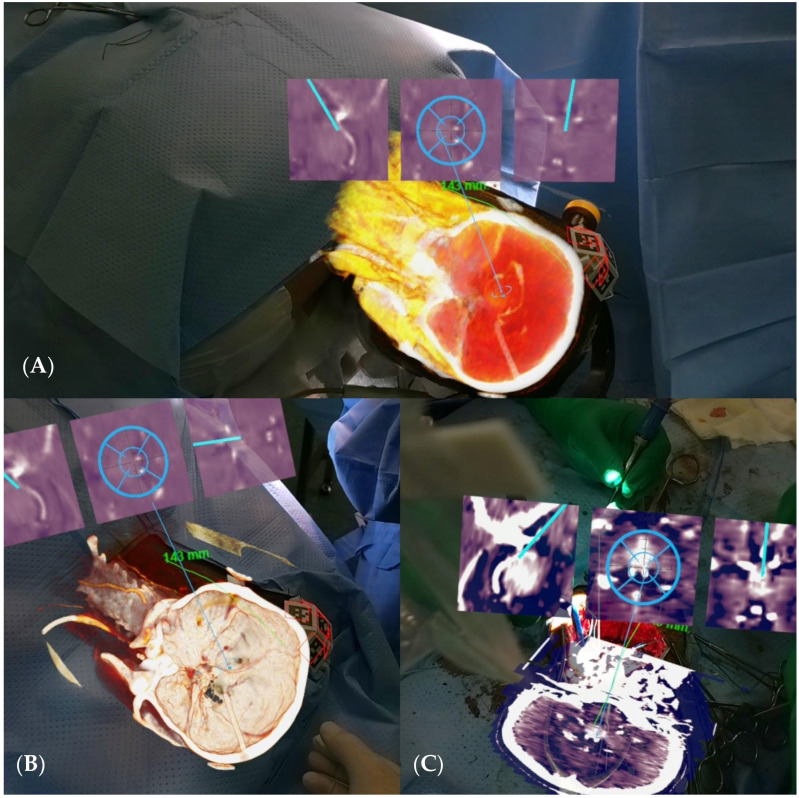
(**A**,**B**) AR view with target on the lesion. (**C**) AR view with target lesion during the surgical procedure.

**Figure 6:**
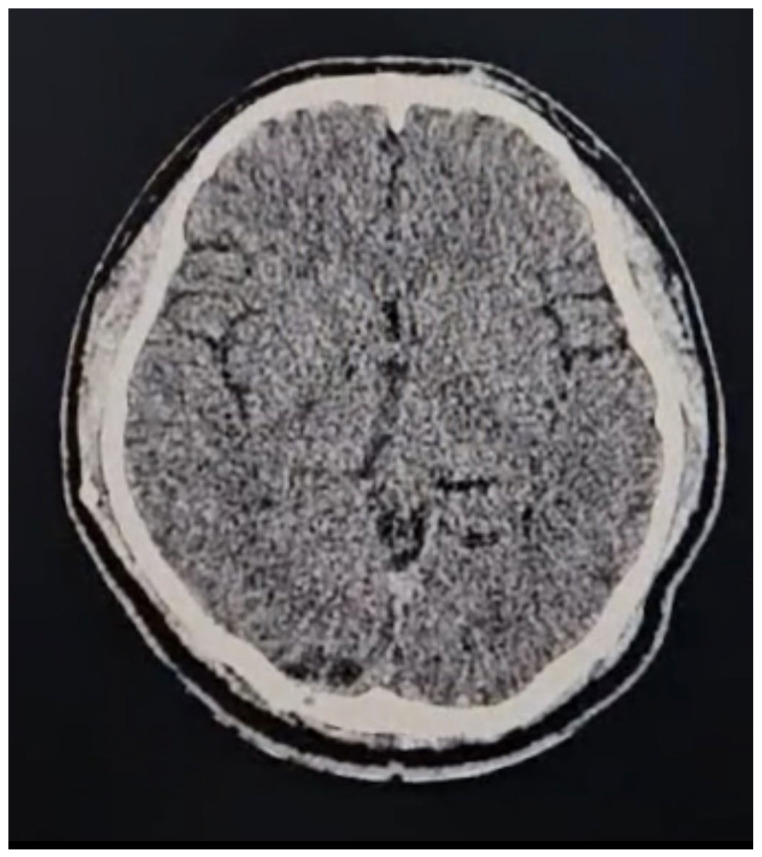
Post-procedure cranial CT scan with no lesion.

A second surgery was performed using a transtentorial approach, assisted by augmented reality (AR) navigation, resulting in the resection of approximately 95% of the tumor (Figure 5A–C). A postoperative CT scan confirmed the surgical changes and near-total resection of the tumor without complications (Figure 6). The patient was discharged after 48 h with a good outcome. Histopathology confirmed the tumor as a grade III ependymoma.

### 3.3. Case 3

**Figure 7:**
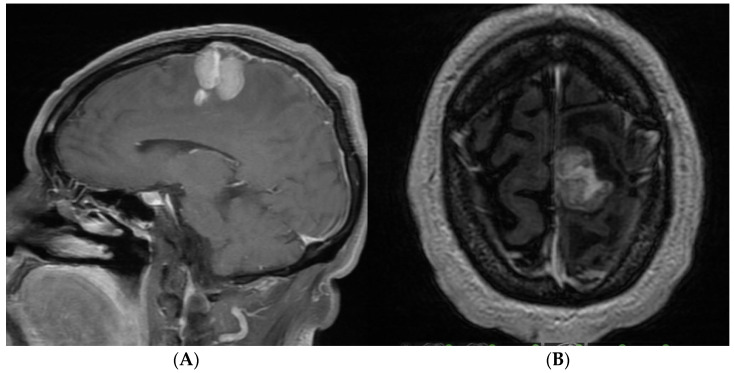
(**A**) Brain MRI sagittal view with homogeneous oval image, multilobulated 1.3 × 2.53 × 3.49 cm. (**B**) Brain MRI axial view with homogeneous oval image, multilobulated 1.3 × 2.53 × 3.49 cm.

A 68-year-old male with a history of arterial hypertension presented with focal seizures, characterized by clonic movements of the right arm and right hemiparesis. During his consultation, a contrast-enhanced MRI of the brain revealed a homogeneous, multilobulated, oval lesion measuring 1.3 × 2.53 × 3.49 cm. The lesion was T1 hypointense, T2 hyperintense with perilesional edema, and showed contrast enhancement in the pre-rolandic right hemisphere (Figure 7A,B).

**Figure 8:**
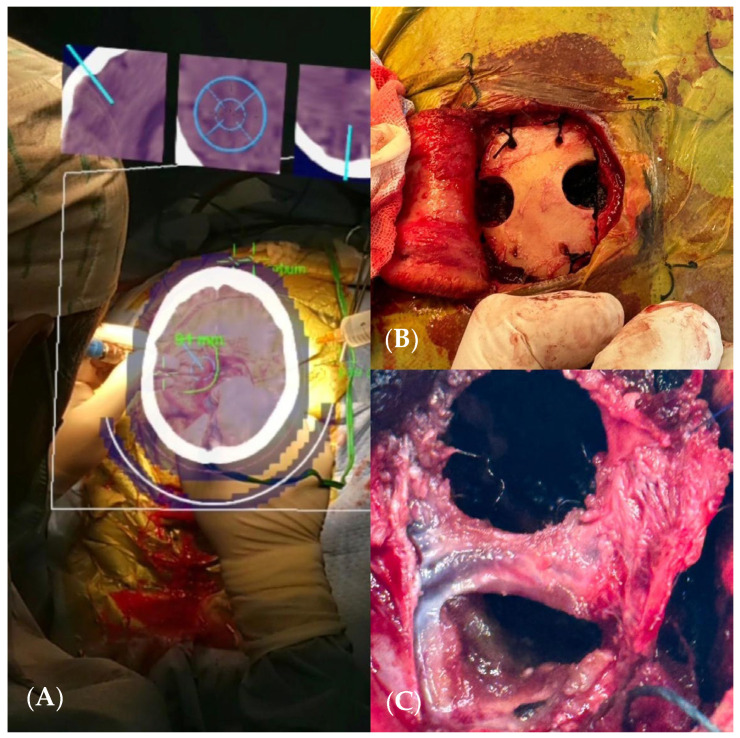
(**A**) AR view showing a real-time CT scan. (**B**) Craniectomy assisted by augmented reality. (**C**) Total resection of the lesion.

**Figure 9:**
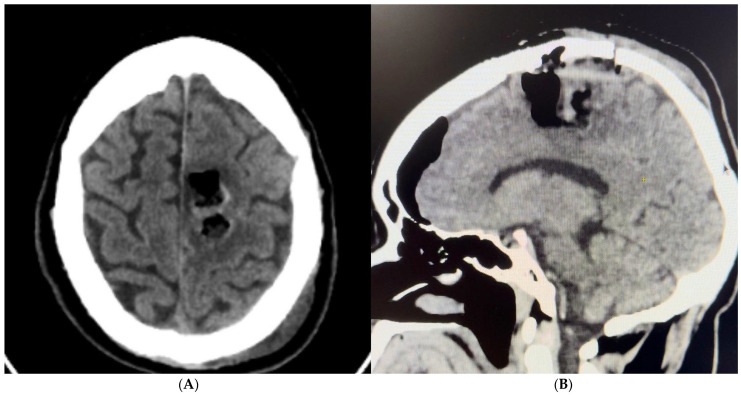
(**A**,**B**) Postoperative CT scan confirmed surgical changes and complete resection of the lesion.

A craniectomy assisted by augmented reality (AR) navigation was performed, achieving total resection of the lesion (Figure 8A–C). A postoperative CT scan confirmed surgical changes and complete resection of the lesion (Figure 9A,B). The patient had an excellent outcome, with no disabilities and complete recovery of movement on the right side of his body, and they remained seizure-free.

Patients were monitored postoperatively for any complications. Follow-up included clinical assessments and postoperative imaging (MRI or CT) to evaluate the extent of resection and detect any residual disease.

Data were collected on patient demographics, clinical presentation, imaging findings, surgical details, intraoperative AR/MR usage, and postoperative outcomes. The accuracy of AR/MR navigation, the duration of surgery, the extent of resection, and postoperative recovery were analyzed to assess the effectiveness of AR/MR technologies in cranial surgery.

The study was conducted in accordance with the Declaration of Helsinki. Informed consent was obtained from all patients, and ethical approval was granted by the hospital’s institutional review board.

Descriptive statistics were used to summarize the data. The accuracy of AR/MR-assisted navigation was evaluated by comparing preoperative planning and intraoperative findings. Postoperative outcomes were assessed using clinical and imaging follow-up data.

## 4. Discussion

This pioneering study represents the first documented use of augmented reality (AR) and mixed reality (MR) in cranial surgery within Latin America, marking a significant milestone in the integration of advanced technology into neurosurgical practice. The importance of this work lies in describing the use and application in real surgery of a technology that allows us the same accuracy of conventional navigators, applied for surgery, pre- and trans-operative times, planning, taking decisions in real time, and making changes if needed, with greater portability of the technology and without the need for additional equipment that interferes in the surgical field. Our results demonstrate the feasibility, accuracy, and clinical benefits of utilizing AR and MR technologies in complex cranial surgeries, offering a paradigm shift in surgical precision and patient outcomes [9,11].

### 4.1. Technological Integration and Surgical Precision

The application of AR and MR in neurosurgery enhances the surgeon’s ability to visualize and navigate intricate anatomical structures. By overlaying 3D digital information directly onto the surgical field, these technologies provide real-time guidance, which significantly improves the accuracy of tumor localization and resection. This capability was particularly evident in the cases presented, where AR-assisted navigation allowed for precise tumor resection with minimal disruption to surrounding critical structures. Our study utilized VisAR software integrated with Microsoft HoloLens 2, demonstrating the effectiveness of this combination in enhancing surgical precision. Other studies have explored different AR systems and reported similar benefits [12,13]. For example, Scherschinski et al. (2022) used a brain contrast MRI scan obtained after endovascular embolization that was then loaded into the frameless stereotactic neuronavigation system (BrainLab Curve). Using the BrainLab SmartBrush software (BrainLab version 3.0), segmentation of the draining veins, feeder arteries, and eloquent structures was carried out on the 3D model by assigning different colors, finding that AR significantly improved the visualization of vascular models, thereby enhancing surgical accuracy and outcomes [14]. These findings suggest that ongoing advancements in AR and MR technologies will continue to improve their efficacy and broaden their applications in surgical practice [15]. In our study, AR-assisted the navigation allowed for precise tumor localization and resection, significantly enhancing the surgeon’s ability to navigate complex anatomical structures. This finding is consistent with other studies that have reported similar benefits. For instance, Encarnacion et al. (2024) observed that AR support in spinal surgery led to significant improvements in accuracy and efficiency, highlighting the potential of AR to enhance surgical outcomes across different types of surgeries [11].

### 4.2. Case Outcomes and Clinical Benefits

The three cases detailed in this study highlight the diverse applications and benefits of AR and MR in neurosurgical procedures:

1. Case 1: The AR-assisted retrosigmoid approach facilitated an 80% subtotal resection of a complex infratentorial meningioma, resulting in significant symptomatic relief and minimal postoperative complications. This underscores the potential of AR to enhance surgical outcomes in challenging anatomical regions (on the sigmoid and transverse sinuses). (Figure 1 and Figure 2). The patient’s rapid recovery and favorable outcome further emphasize the clinical advantages of AR-guided surgery.
2. Case 2: The AR-assisted transtentorial approach enabled near-total resection of a pineal region ependymoma, illustrating the technology’s efficacy in managing deep-seated tumors with complex vascular relationships; guided by AR to the pineal region where the tumor was located, in turn, AR allowed us to know the limits of the tumor; those limits were blocked to direct vision by the parenchyma and vascular structures (in-ferior sagittal sinuses, internal cerebral veins, basal of Rosenthal, vein of Galen, rectus, and inferior longitudinal sinus).
3. Case 3: The use of AR in guiding craniectomy for a pre-rolandic lesion ensured complete resection with excellent functional recovery, demonstrating the precision and effectiveness of AR in locate cortical tumor and the main benefit was knowing the exact topographic relationship of vascular structures where we performed a classic craniotomy and posterior interhemispheric dissection preventing the risk of an inadvertent vascular lesion.

### 4.3. Clinical Outcomes

The positive clinical outcomes observed in our cases align with those reported in other AR- and MR-assisted surgical studies. Our patients experienced no postoperative complications and favorable recoveries, with significant symptom resolution and excellent functional outcomes. Bocanegra et al. (2024) conducted a systematic review of AR in neurosurgery, noting that AR-assisted procedures resulted in higher accuracy and better patient outcomes compared to traditional methods [16]. This study reinforces our findings, suggesting that AR and MR technologies can improve surgical precision and reduce operative risks. All of the procedures had a gross total resection of 95%.

### 4.4. Comparison with Conventional Techniques

AR and MR technologies offer several advantages over traditional surgical navigation systems. The enhanced spatial awareness and real-time feedback provided by AR reduce the risk of intraoperative errors and improve surgical efficiency. Studies have shown that AR-assisted surgeries result in higher accuracy and better outcomes compared to conventional methods. Our findings align with these reports, as evidenced by the successful outcomes in all three cases [10,17,18].

### 4.5. Challenges and Considerations

While the benefits of AR and MR in neurosurgery are substantial, several challenges must be addressed for broader adoption. These include the need for specialized training to ensure proficiency with AR systems, the initial costs associated with acquiring and implementing the technology, and the integration of AR systems into existing surgical workflows. Future time indents should focus on enhancing the user interface, reducing the setup time, and improving the accuracy and reliability of AR systems [19,20].

### 4.6. Cost-Effectiveness and Accessibility

The initial costs associated with acquiring and implementing AR and MR technologies can be substantial. However, the long-term benefits, including improved surgical precision, reduced operative time, and enhanced patient outcomes, may offset these initial expenses. Krause et al. (2024) highlighted the potential cost-effectiveness of AR in surgery, suggesting that the technology’s ability to reduce complications and improve outcomes could lead to overall cost savings in the healthcare system. Our findings align with this perspective, as the improved surgical outcomes observed in our cases suggest that AR and MR technologies could offer a cost-effective solution for complex cranial surgeries [21,22].

### 4.7. Learning Curve

AR technology has recently gained renewed interest, particularly in neurosurgery [23]. Though still in its early stages of adoption in operating rooms worldwide, there is significant enthusiasm for its potential. However, integrating AR systems into surgical procedures presents challenges, both preoperatively and intraoperatively. Urakov et al. [24] documented an unexpected shutdown of AR software 1.0, leading to prolonged surgery times. The complexity of AR spinal navigation (ARSN) and advanced augmented display head-mounted devices (AD-HMDs) can also deter seasoned surgeons from embracing these technologies in their practice [25]. Evidence shows that the consistent use of AR improves surgical techniques and patient outcomes. Gasco et al. [26] found that AR as an educational tool reduced errors by nearly 50% compared to traditional training methods. Moreover, using AR in neurosurgery education could shorten training periods, allowing healthcare institutions to train more neurosurgeons efficiently and gain financial advantages [27,28,29,30].

### 4.8. Looking Ahead

As AR technology advances, its potential applications in neurosurgery are both broad and diverse. Future developments may include more advanced AI integration, enabling even more precise surgical planning and real-time decision-making support. Additionally, the creation of haptic feedback systems could enhance the tactile experience in virtual environments. The fusion of 3D printing with augmented reality (AR) in spinal surgery marks a significant shift in how procedures are planned and executed. By using patient-specific 3D-printed models, surgeons gain access to accurate, tangible replicas of patient anatomy, which significantly enhances both preoperative planning and intraoperative guidance [31,32,33,34].

When combined with AR, these 3D-printed models can be overlaid with dynamic digital data, such as nerve pathways and vascular structures. This integration improves surgeons’ understanding and planning accuracy, allowing for a thorough assessment of the patient’s unique anatomy [35]. During surgery, AR projects this enhanced information directly into the surgeon’s field of view, enabling real-time comparisons between the model and the actual surgical site. This capability is especially crucial for tasks requiring high precision, like screw placement or custom implant insertion [36,37,38,39].

Recent innovations have documented the use of AR with 3D-printed models in spinal surgeries, from enhanced preoperative planning to guiding surgical procedures and postoperative assessments that compare actual outcomes with initial plans [40,41,42].

Looking forward, there is significant potential for technological advancements to further strengthen the synergy between 3D printing and AR [43]. Areas ripe for development include automated adjustments to models based on real-time feedback, integration with machine learning to optimize surgical strategies, and enhancements in training programs for new surgeons [44,45]. These advancements could streamline surgical procedures and improve success rates, profoundly impacting both surgical outcomes and the training of future surgeons [46,47]. However, the high cost of implementing and maintaining advanced AR systems remains a significant obstacle. This financial barrier may restrict access primarily to well-funded healthcare institutions, potentially slowing the widespread adoption of AR in clinical practice despite its potential to revolutionize surgical procedures and educational methods [48,49,50,51,52,53].

### 4.9. Limitations and Future Directions

1. Limited Case Sample: The study only presents three case studies, which may not be sufficient to generalize the effectiveness and reliability of AR and MR technologies in cranial surgery across diverse patient populations and different types of cranial conditions.
2. Short-Term Follow-Up: The article primarily discusses immediate postoperative outcomes, with no information on long-term follow-up. Long-term data are crucial to assess the durability of surgical outcomes and the potential for delayed complications.
3. Lack of a Control Group: The study does not include a control group undergoing traditional surgical navigation methods. This omission makes it difficult to directly compare the benefits and potential drawbacks of AR/MR-assisted surgeries versus conventional techniques.
4. Training and Expertise: The article does not provide detailed information on the level of training and expertise required for surgeons to effectively use AR and MR technologies. The learning curve and proficiency levels of different surgeons could significantly impact the outcomes.
5. Cost Analysis: There is a lack of detailed cost analysis comparing AR/MR-assisted surgeries to conventional methods. Understanding the financial implications, including initial setup costs, maintenance, and potential savings from improved outcomes, is essential for broader adoption.
6. Technological Limitations: The study acknowledges the technological setup and accuracy (2–3 mm) but does not discuss potential technical failures, software glitches, or the impact of hardware limitations in real-time surgical environments.
7. Subjective Assessments: The reported benefits, such as enhanced precision and better outcomes, are largely qualitative and based on the authors’ observations. More objective metrics and standardized assessment tools would strengthen the evidence for AR and MR technologies in cranial surgery.
8. Potential Bias: The involvement of the authors in pioneering the use of these technologies could introduce bias. Independent studies by other researchers or institutions would be valuable to corroborate the findings and minimize potential bias [54].

The future of AR and MR in neurosurgery lies in their integration with other modalities, such as intraoperative imaging and robotic assistance, to further enhance surgical precision and outcomes [55]. The development of low-cost, user-friendly AR systems will be crucial for their widespread adoption in clinical practice, making advanced surgical navigation accessible to a broader range of healthcare providers and patients [56,57,58,59].

## 5. Conclusions

This pioneering study showcases the first documented use of augmented reality (AR) and mixed reality (MR) in cranial surgery in Latin America, conducted at Hospital Ángeles Universidad in Mexico City. The integration of AR and MR technologies significantly enhanced surgical precision and clinical outcomes by providing real-time, 3D digital overlays during complex neurosurgical procedures.

The three case studies presented demonstrate the diverse applications and benefits of AR and MR, resulting in successful outcomes with minimal postoperative complications. The use of VisAR software with Microsoft HoloLens 2 proved effective in improving tumor localization and resection accuracy while reducing disruption to critical structures. Despite the promising results, challenges such as the need for specialized training, high costs, and integration into existing workflows must be addressed for wider adoption. Future research should focus on long-term outcomes, larger sample sizes, and comparative studies to validate the efficacy and cost-effectiveness of AR and MR in neurosurgery. Continued advancements and improvements in these technologies are expected to enhance their clinical utility and adoption in neurosurgical practices worldwide.

## Data Availability

The original contributions presented in the study are included in the article, further inquiries can be directed to the corresponding author/s.

## Abbreviations

AR, augmented reality; CT, computed tomography; MRI, magnetic resonance imaging; ER, Emergency Room; MR mixed reality; VR, virtual reality; GTR, gross total resection.
